# Supplementary material for: Skin immunisation activates an innate lymphoid cell-monocyte axis regulating CD8+ effector recruitment to mucosal tissues
Source: Nat Commun. 2019 May 17;10:2214. doi: 10.1038/s41467-019-09969-2 (PMC6525176; doi:10.1038/s41467-019-09969-2)
Supplement: Supplementary file 1 — Supplementary Information [file 41467_2019_9969_MOESM1_ESM.pdf]

Supplementary Information

Skin immunisation activates an innate lymphoid cell-monocyte axis regulating CD8$^+$ effector recruitment to mucosal tissues.

Zaric et al.

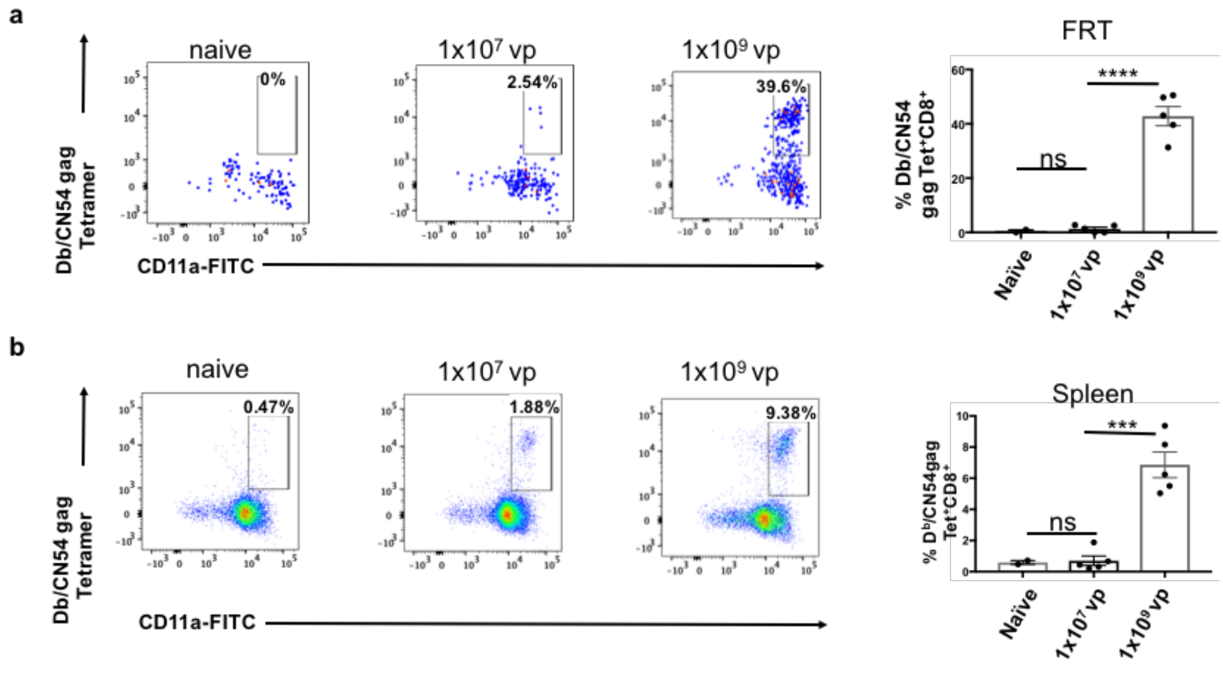

Supplementary Figure 1.

Tetramer$^+$CD8$^+$ T cell responses in the FRT and spleen after skin immunisation with a dose titration of Ad CN54gag. Mice were immunised ID with 1 X 10^9 or 1 X 10^7 vp of Ad-CN54gag. Representative flow cytometry dot-plots and summary graphs showing frequency of D^b/CN54 gag Tet$^+$ CD8$^+$ T cells from the FRT (**a**) and spleen (**b**) on day 14-post immunisation and naïve controls. Data represent mean ± SEM of n=5 /experimental groups and n=2 naïve. ***p<0.001 by one-way ANOVA.

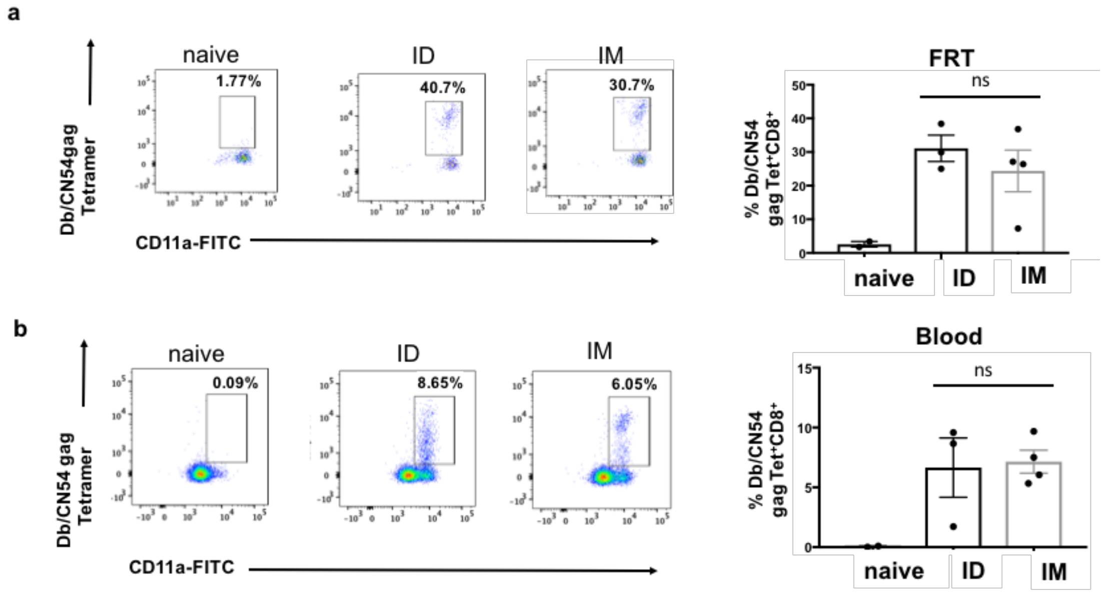

Supplementary Figure 2.

Immunisation by the ID and IM route recruit CD8$^+$ T cells to the FRT. Representative flow cytometry plots and summary graphs showing frequencies of CD8$^+$ D^b/CN54 gag Tet$^+$ cells from (**a**) FRT tissue and (**b**) blood on day 14 after ID or IM immunisation with Ad-CN54gag and from naïve mice. Data represent mean ± SEM of n=3-4 /experimental groups and n=2 naive. Data were analysed by one-way ANOVA, ns not significant.

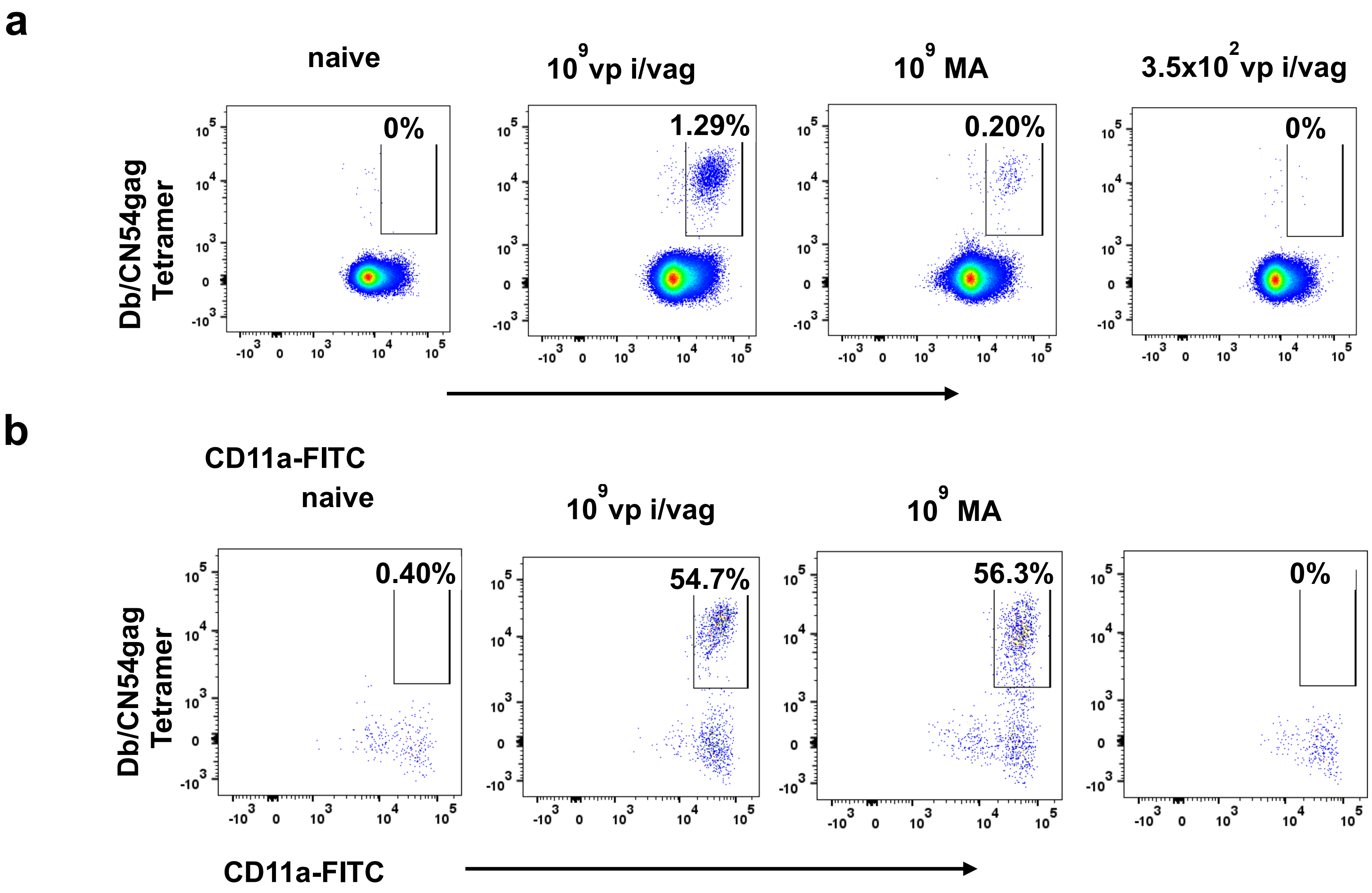

Supplementary Figure 3.

Ad vaccine bio-distributed to FRT does not prime CD8$^+$ T cells in FRT or iliac LNs. Mice were immunised by MA into skin or by injection into the vaginal mucosa (i/vag) with 1x10^9 vp Ad-CN54gag. Some mice received 3.5x10^2 vp of the same vaccine by intra-vaginal injection. Representative flow cytometry dot-plots showing frequency of Db/CN54 gag tet$^+$ CD8$^+$ T cells in the iliac LNs (**a**) and FRT tissue (**b**) on day 14-post immunisation. Data are representative of three independent experiments, n=7-9 / group.

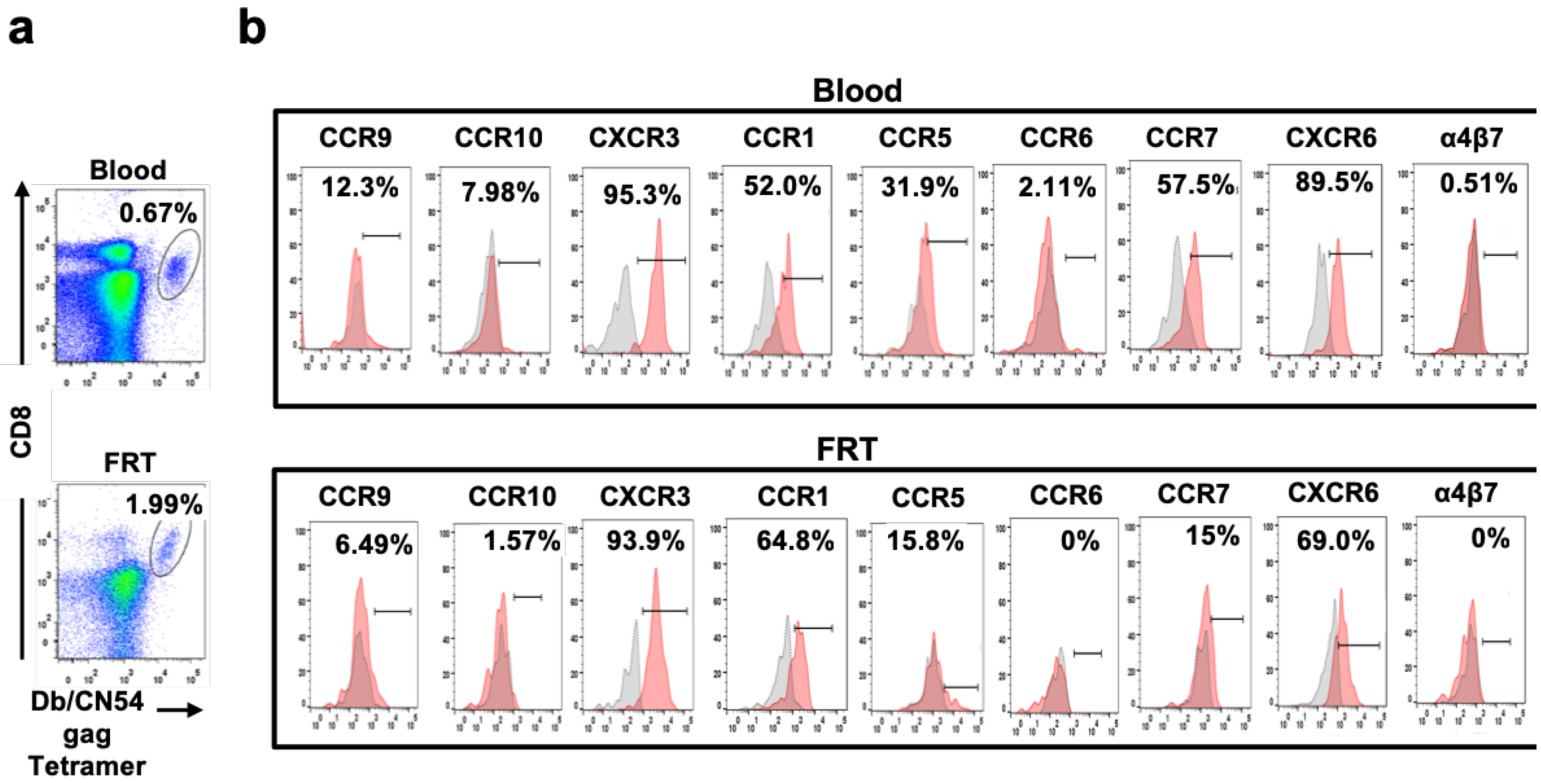

Supplementary Figure 4.

Chemokine receptor and integrin expression on antigen–specific $CD8^+$ T cells present in the FRT after skin immunisation. (**a**) Representative flow cytometric dot plots of D^b/CN54 gag^+ $CD8^+$ T cells from the peripheral blood (upper panel) and FRT (lower panel) on day 7 after immunisation with Ad-CN54 gag ($1x10^9$ vp) by MA to the skin. (**b**) Representative histograms showing expression of CCR9, CCR10, CCR1, CCR5, CCR6, CCR7, CXCR3, CXCR6 and **α**4β7 (gated on D^b/CN54 gag tet^+ $CD8^+$ cells) from peripheral blood (upper panel) or FRT tissue (lower panel) on day 7 after skin MA immunisation with Ad-CN54 gag. MA (red histograms); grey-filled histograms represent unstained control. Numbers within histograms indicate percent chemokine / integrin receptor expressing cells. Data are representative of two independent experiments.

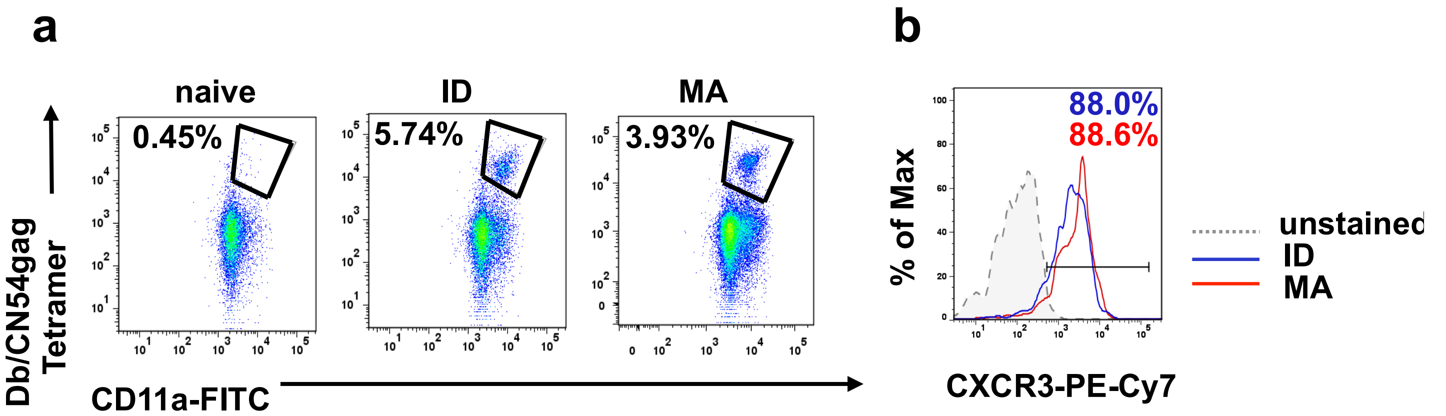

Supplementary Figure 5.

Antigen-specific CD8^{+} T cells recruited to the lung after skin immunisation express CXCR3.

Mice were immunized with Ad-CN54 gag either by ID injection or MA skin application or left naive. Lungs were harvested 14 days post immunisation. (**a**) Representative flow cytometry histograms showing frequency of D^{b}/CN54gag tetramer^{+} cells (gated on CD45^{+}CD4^{-},MHCII^{-}CD8^{+} cells) in the lung induced by either MA or ID immunisation. Data are representative of 3 independent experiments (n=6-9 per group). (**b**) Representative histograms indicating CXCR3^{+} profile of gated D^{b}/CN54 gag tetramer^{+} CD8^{+} T cells in lung tissue of MA (red histogram) or ID (blue histogram) immunised mice. Grey-filled histograms represent unstained control immunised mouse.

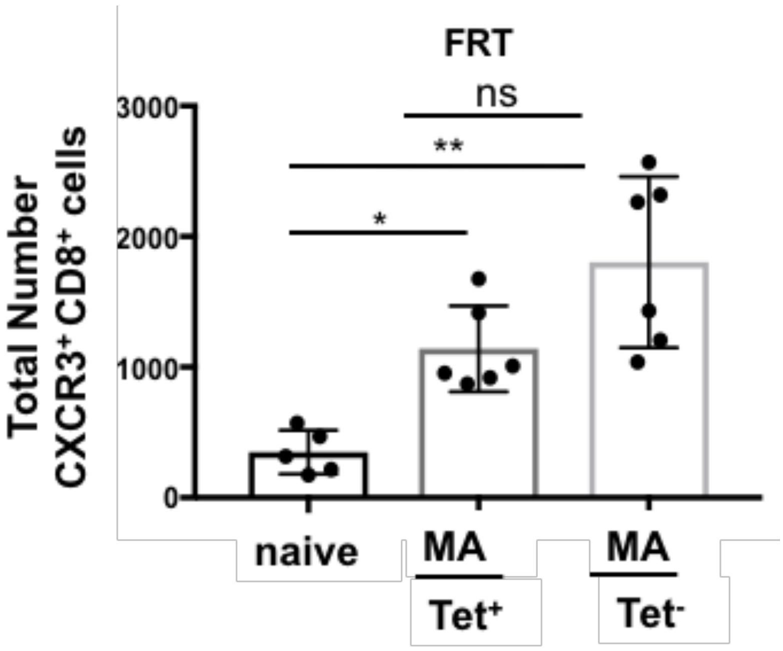

Supplementary Figure 6.

CXCR3 is expressed by CD8^{+} T cells recruited to the FRT. Total numbers of CXCR3^{+} CD8^{+} cells in the FRT of naïve and Ad CN54gag immunised mice were analysed by flow cytometry at day 14-post immunisation. Bar graph summarises total number of CXCR3^{+} cells from a gated CD45^{+}CD8^{+}D^{b}/CN54gag tetramer negative (Tet^{-}) or tetramer positive (Tet^{+}) population from two independent experiments (n=6 for experimental groups). * $p<0.05$, **$p<0.001$ by one-way ANOVA.

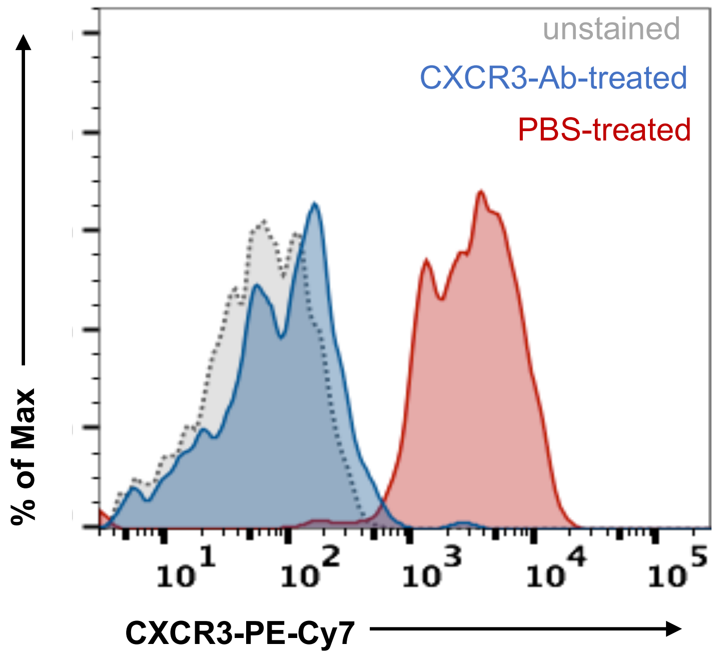

Supplementary Figure 7.

Anti-CXCR3 mAb blocks CXCR3 on effector OT-I cells. Congenic CD45.1 OT-I cells (2×10^5) were transferred into WT mice. Five days after Ad-OVA skin immunisation some were treated with anti-CXCR3 Ab. On day 7, effector OT-I cells (2×10^6) isolated from these mice were transferred into secondary hosts (some treated with anti-CXCR3 Ab) infected with either Ad-OVA via MA or intra-vaginal injection 3.5 days earlier. Some secondary recipients were intra-vaginally injected with Ad-CN54 gag or PBS. 3.5 days prior to effector OT-I cells transfer. One and a half day after adoptive transfer of the effector OT-I cells into secondary recipients, CXCR3 expression on CD45.1$^+$ OT-I cells isolated from the spleens of different recipients was assessed by flow cytometry. Flow cytometry histograms, representative from two independent experiments (n=6 per experimental group) are shown.

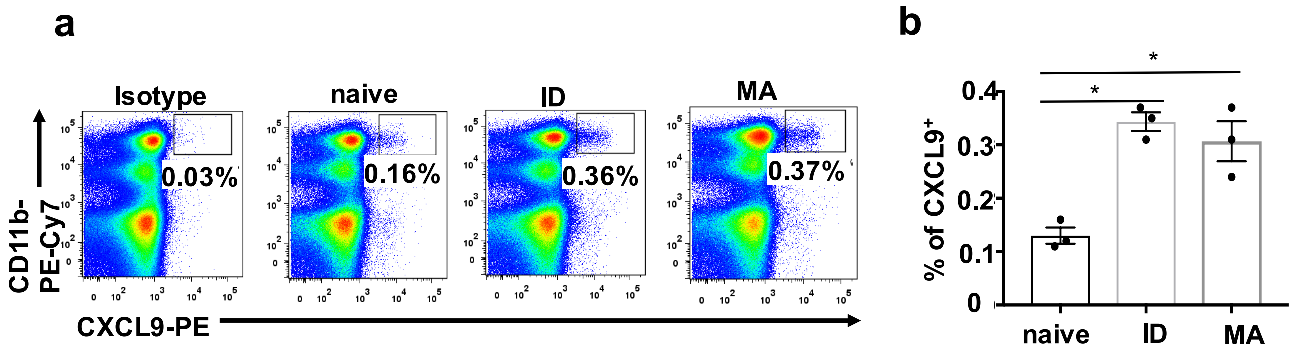

Supplementary Figure 8.

CD11b$^+$ cells in the respiratory tract express the CXCL9 ligand for CXCR3$^+$ cells after skin immunization. (a) Representative flow cytometry histograms and **(b)** summary graph showing frequency of CD11b$^+$ cells expressing CXCL9 (gated on CD45$^+$) from lungs of ID or MA immunised mice at day 3 and naïve controls. Data are representative of three independent experiments, n=3 per group. * $P < 0.05$, by one-way ANOVA, error bars show s.e.m.

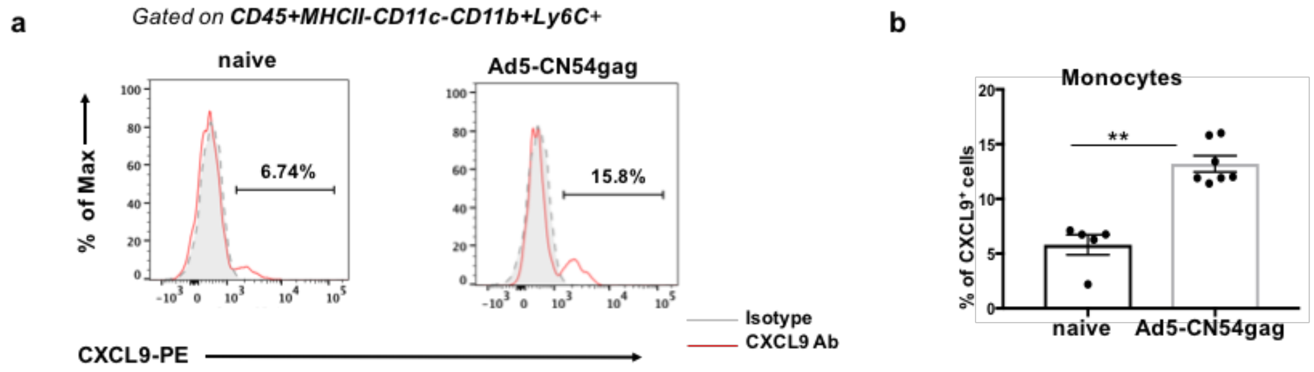

Supplementary Figure 9.

Monocytes infiltrating the FRT express the CXCR3 ligand CXCL9 after skin immunisation. (**a**) Representative flow cytometry histograms and (**b**) summary bar graph of CXCL9 expressed on $CD45^{+}Ly6C^{+}CD11b^{+}MHCII^{-}CD11c^{-}$ cells isolated from FRT tissue at day 14 after skin immunisation with Ad-CN54 gag via ID injection or from naïve mice. Data represent mean ± SEM of n=5-7 /experimental group. **p<0.01 by one-way ANOVA.

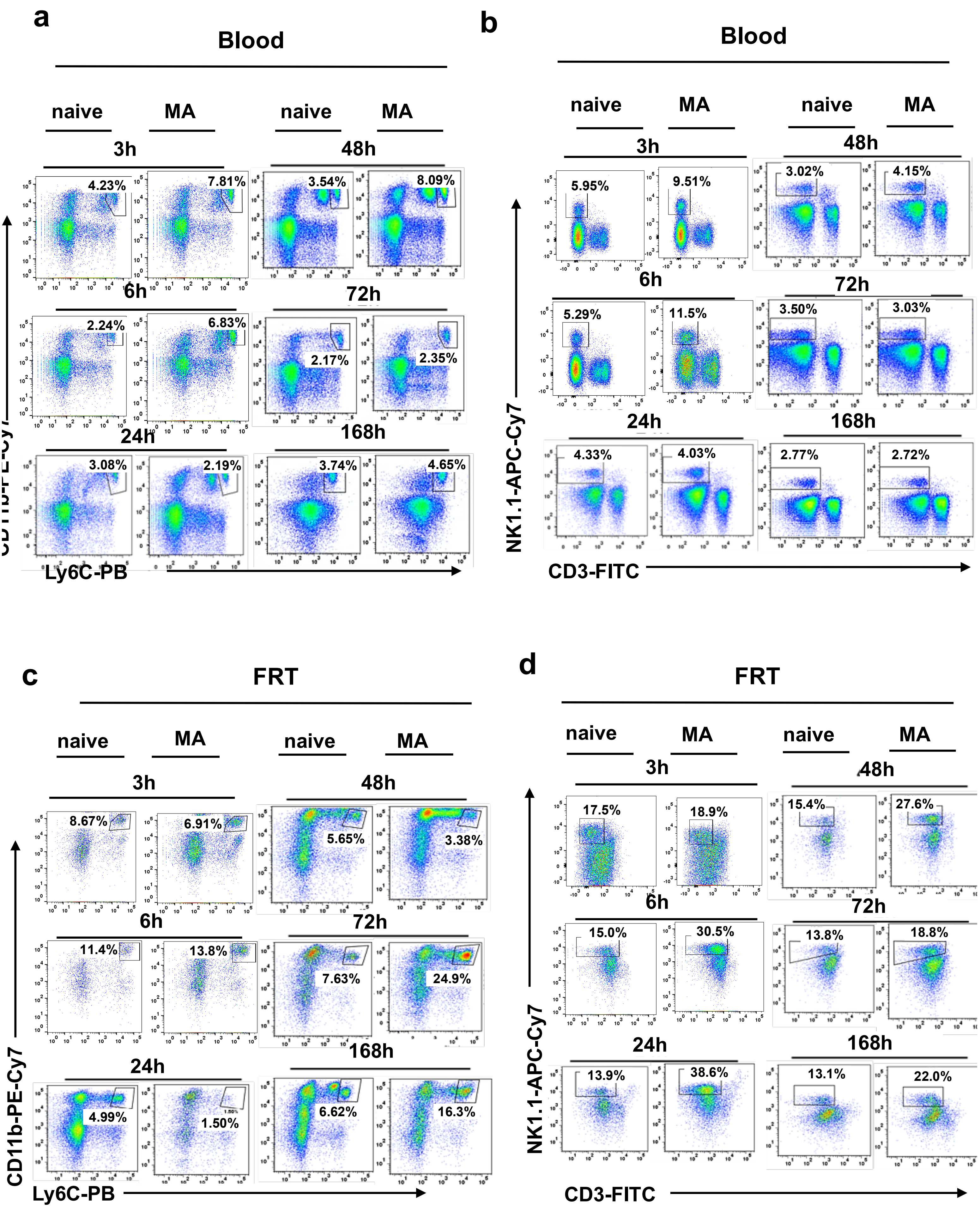
a
Blood
naive
MA
naive
MA
3h
48h
4.23%
7.81%
3.54%
8.09%
6h
72h
2.24%
6.83%
2.17%
2.35%
24h
168h
3.08%
2.19%
3.74%
4.65%
Ly6C-PB
b
Blood
naive
MA
naive
MA
3h
48h
5.95%
9.51%
3.02%
4.15%
6h
72h
5.29%
11.5%
3.50%
3.03%
24h
168h
4.33%
4.03%
2.77%
2.72%
NK1.1-APC-Cy7
CD3-FITC
c
FRT
naive
MA
naive
MA
3h
48h
8.67%
6.91%
5.65%
3.38%
6h
72h
11.4%
13.8%
7.63%
24.9%
24h
168h
4.99%
1.50%
6.62%
16.3%
CD11b-PE-Cy7
Ly6C-PB
d
FRT
naive
MA
naive
MA
3h
48h
17.5%
18.9%
15.4%
27.6%
6h
72h
15.0%
30.5%
13.8%
18.8%
24h
168h
13.9%
38.6%
13.1%
22.0%
NK1.1-APC-Cy7
CD3-FITC

Supplementary Figure 10.

Skin immunisation with Ad-CN54 gag promotes the early recruitment of group 1 ILCs and monocytes to the FRT. Representative flow cytometry dot plots of monocytes ($Ly6C^{+}CD11b^{+}$) (**a,c**) and group 1 ILCs ($NK1.1^{+}CD3^{-}$) (**b,d**) in peripheral blood or FRT tissue at indicated time points after skin immunisation by MA with Ad-CN54 gag Data are representative from three independent experiments, n=6-9 per group.

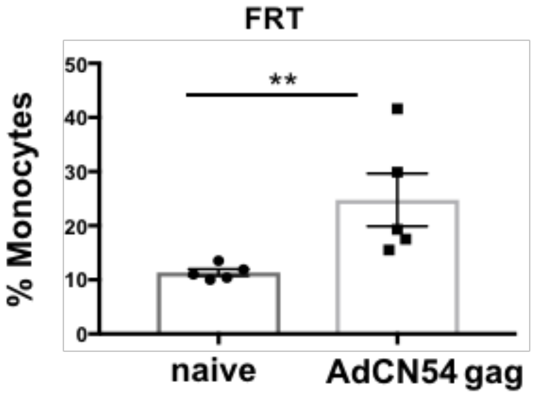

Supplementary Figure 11.

Ly6C$^+$ monocytes remain elevated in the FRT at 14 days after skin immunisation. Bar graph summaries frequency of monocytes (CD45$^+$Ly6C$^+$CD11b$^+$MHCII$^-$) analysed in FRT tissue from naïve or Ad-CN54gag skin immunised mice on day 14. Data represent mean ± SEM of n=5 /experimental group. **p<0.01 by one-way ANOVA.

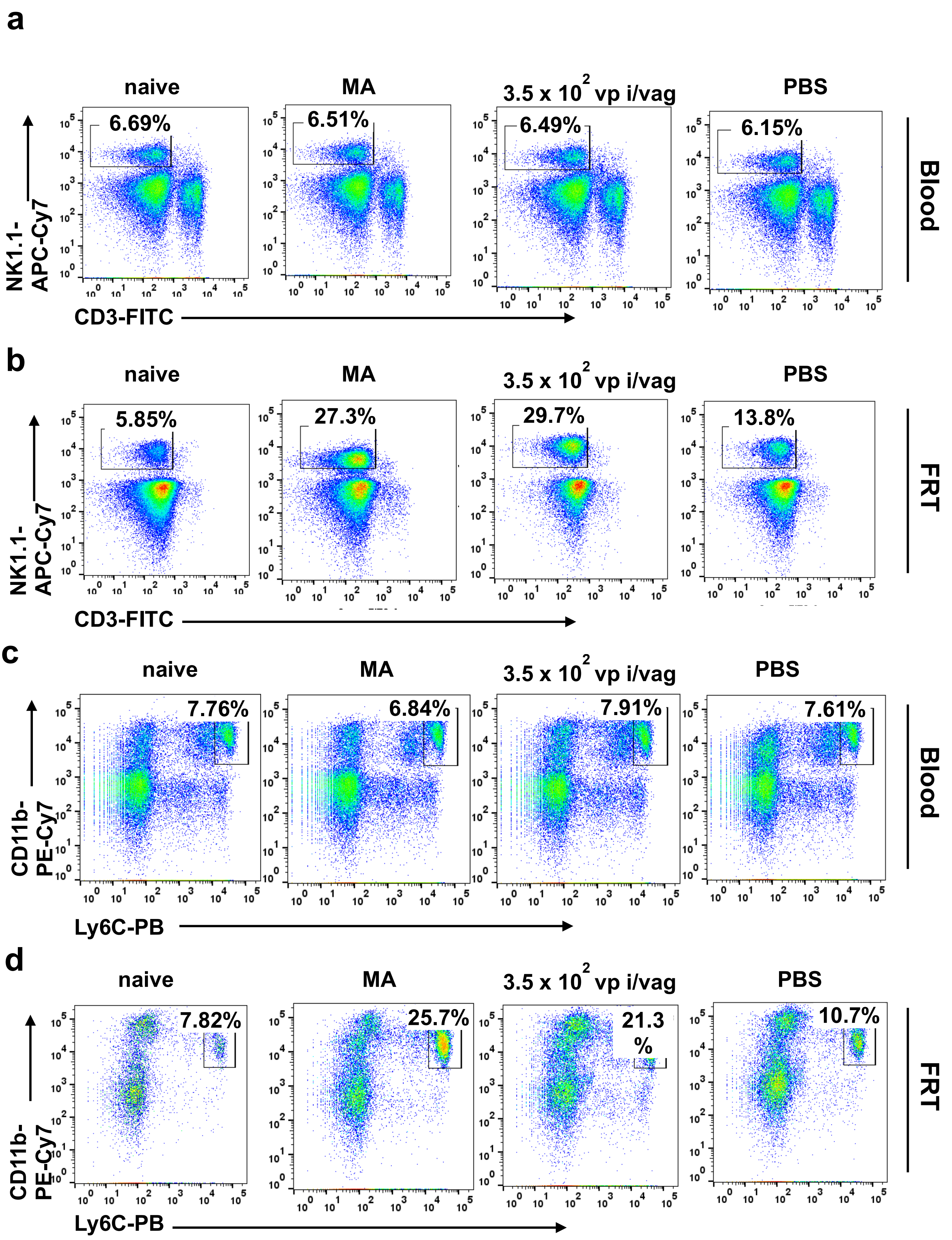
a
naive
MA
3.5 x 10^2 vp i/vag
PBS
6.69%
6.51%
6.49%
6.15%
NK1.1-APC-Cy7
CD3-FITC
Blood
b
naive
MA
3.5 x 10^2 vp i/vag
PBS
5.85%
27.3%
29.7%
13.8%
NK1.1-APC-Cy7
CD3-FITC
FRT
c
naive
MA
3.5 x 10^2 vp i/vag
PBS
7.76%
6.84%
7.91%
7.61%
CD11b-PE-Cy7
Ly6C-PB
Blood
d
naive
MA
3.5 x 10^2 vp i/vag
PBS
7.82%
25.7%
21.3 %
10.7%
CD11b-PE-Cy7
Ly6C-PB
FRT

Supplementary Figure 12.

Intra vaginal injection of equivalent Ad vector dose bio-distributed by MA immunisation induces infiltration of monocytes and group 1 ILCs to the FRT. Mice were immunised with Ad-CN54 gag by MA delivery to skin ($1x10^9$ vp) or by injection to the vaginal mucosa (i/vag) with $3.5x10^2$ vp (equivalent to the bio-distributed Ad vector dose). Control mice were injected PBS (i/vag.). After 1 day (group 1 ILCs) and after 3 days (monocytes) were enumerated from peripheral blood and FRT tissues. Representative flow cytometry dot plots show group 1 ILCs in peripheral blood (**a**), in FRT tissue (**b**), and monocytes in peripheral blood (**c**) and in FRT tissue (**d**). Data are representative of three independent experiments (*n* = 6-9 per experimental group).

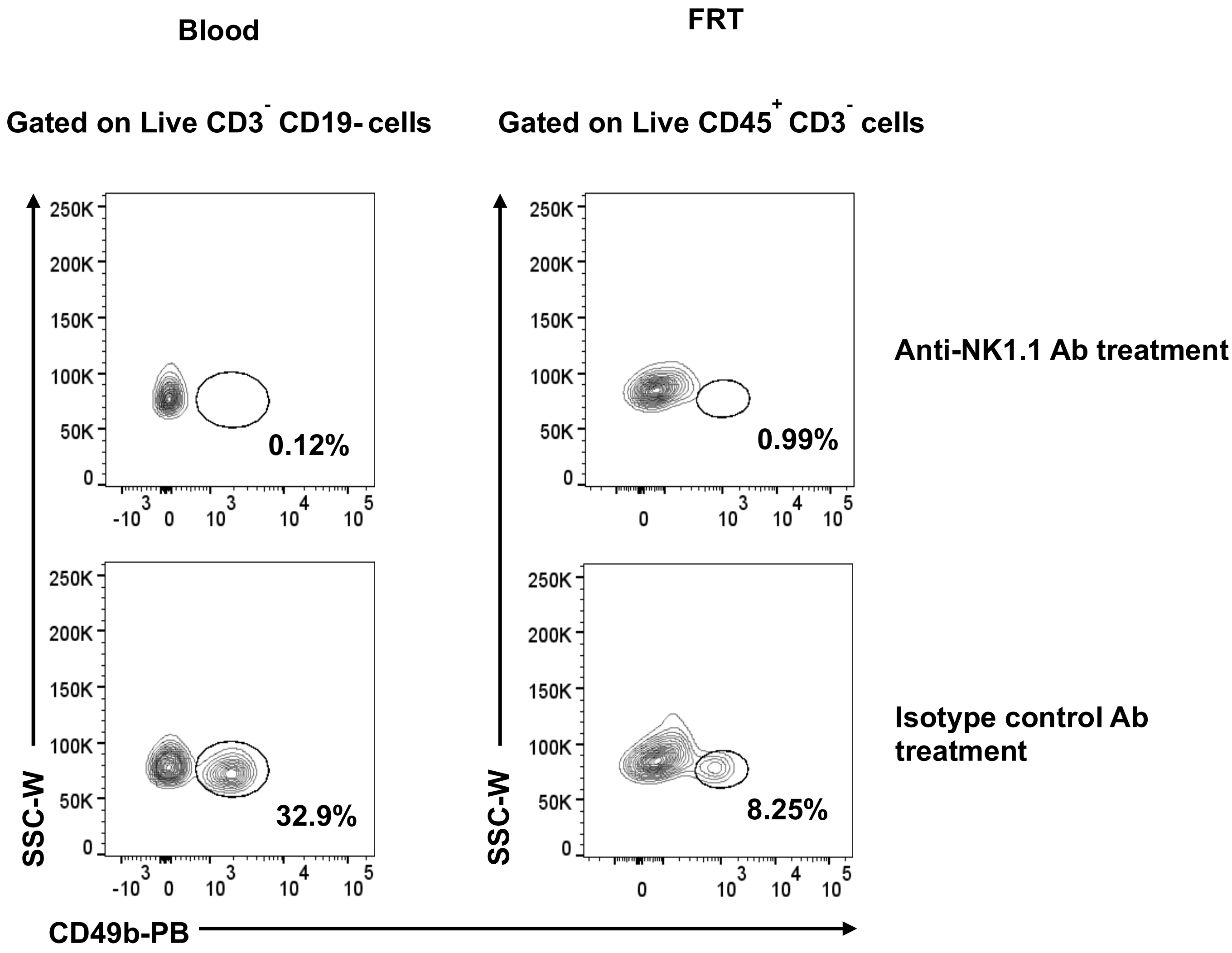

Supplementary Figure 13.

Anti-NK1.1 mAb depletes group 1 ILCs. (**a**) 200μl of anti-NK1.1 Ab (PK136) or isotype control Ab administered i.p. Frequency of CD19-CD3$^-$CD49b$^+$ cells were analysed in blood and CD45$^+$CD3$^-$CD49$^+$ cells in FRT tissues at 24 h post-injection. Data are representative of six mice.

Nucleotide sequence of HIV-1 CN54 gag

atgggcgccagggccagcatcctgaggggcggcaagctggacaagtgggagaagatcaggctgaggcccggcg
gcaagaagcactacatgctgaagcacctggtgtgggccagcagggagctggagaggttcgccctgaaccccggcc
tgctggagaccagcgagggctgcaagcagatcatgaagcagctgcagagcgccctgcagaccggcaccgagga
gctgaggagcctgttcaacaccgtggccacccccctactgcgtgcacaccgagatcgacgtgagggacaccaggga
ggccctggacaagatcgaggaggagcagaacaagatccagcagaagacccagcaggccaaggaggccgacg
gcaaggtgagccagaactaccccatcgtgcagaacctgcagggccagatggtgcaccagcccatcagccccagg
accctgaatgcatgggtgaaggtggtggaggagaaggccttcagccccgaggtgatccccatgttcagcgccctga
gcgagggcgccacccctcaggacctgaacaccatgctgaacaccgtgggcggccaccaggccgccatgcagatc
ctgaaggacaccatcaacgaggaggccgccgagtgggacaggctgcaccccgtgcacgccggccccatcgccc
ccggccagatgagggagcccaggggcagcgacatcgccggcaccaccagcaacctgcaggagcagatcgcct
ggatgaccagcaacccacccgtgcccgtgggcgacatctacaagaggtggatcatcctgggattgaacaagatcgt
gaggatgtacagccccaccagcatcctggacatcaagcagggccccaaggagcccttcagggactacgtggaca
ggttcttcaagaccctgagggccgagcaggccacccagggcgtgaagaactggatgaccgacaccctgctggtgc
agaacgccaaccccgactgcaagaccatcctgagggccctgggccccggcgccagcatcgaggagatgatgacc
gcctgccagggcgtgggcggccccagccacaaggccaaggtgctggccgaggccatgagccagaccaacagc
gccatcctgatgcagaggagcaacttcaagggcagcaagaggatcgtgaagtgcttcaactgcggcaaggagggc
cacatcgccaggaactgcagggcccccaggaagaagggctgctggaagtgcggcaaggagggccaccagatg
aaggactgcaccgagaggcaggccaacttcctgggcaagatctggcccagccacaagggcggccccggcaactt
cctgcagaacaggcccgagcccaccgcccccccgaggagagcttcaggttcgaggaggagaccaccacccccc
agccagaagcaggagcccatcgacaaggagctgtacccctgaccagcctgaagagcctgttcggcaacgaccc
cagcagccag

Supplementary Figure 14.

Nucleotide sequence of the optimised HIV-1 CN54 gag gene used for cloning into VV.

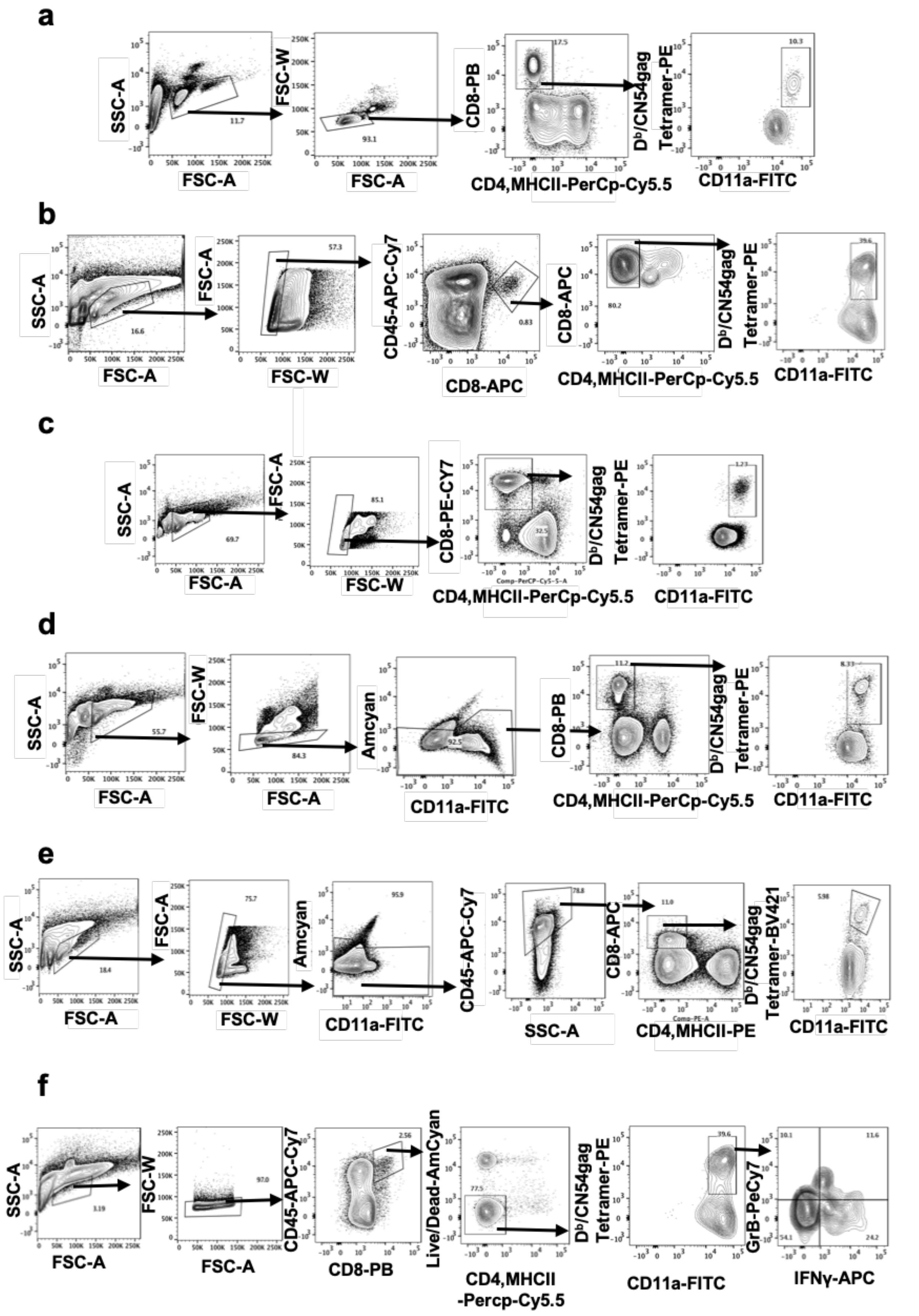

Supplementary Figure 15.

Supplementary Figure 15

Gating strategies used to identify Tet$^+$ cells. (**a,c**) An example of a gating strategy applied to examine Tet$^+$ cells in blood of immunized mice in Figures 1b, 4a (upper panel), Supplementary Figure 2b and LNs in Supplementary Figure 3a. Lymphocytes were gated based on FSC-A and SSC-A and doublets were excluded based on the FSC-W and FSC-A gate. Tet$^+$ cells were identified within CD8$^+$CD4$^-$ MHCII$^-$ gate as a D^b/CN54gag Tet$^+$CD11a^{high} population. **(b)** Additional gating was applied to identify CD45$^+$ cells when Tet$^+$ cells were examined in peripheral tissues, such as FRT, in Figure 1f and 4a (lower panel) and Supplementary Figures 1a, 2a, 3b. **(d,e)** In spleen and lung cell suspensions, auto-fluorescent cells were gated out using an unstained channel (AmCyan). **(e)** Gating strategies used for detection of Tet+ cells in spleens in Supplementary Figure 1b and in lungs in Supplementary figure 5a are shown. (**f**) Gating strategy used to identify poly-functional Tet$^+$ cells in the FRT in Figure 2d is shown. After gating on lymphocytes and single cells based on the SSC-A, FSC-A and FCS-W, CD45$^+$CD8$^+$ cells were selected. Following exclusion of dead cells and cells expressing CD4 and MHCII, Tet$^+$ cells, identified as a D^b/CN54gag Tet$^+$CD11a^{high} population, were further examined for the expression of IFNγ and Granzyme B (GrB).

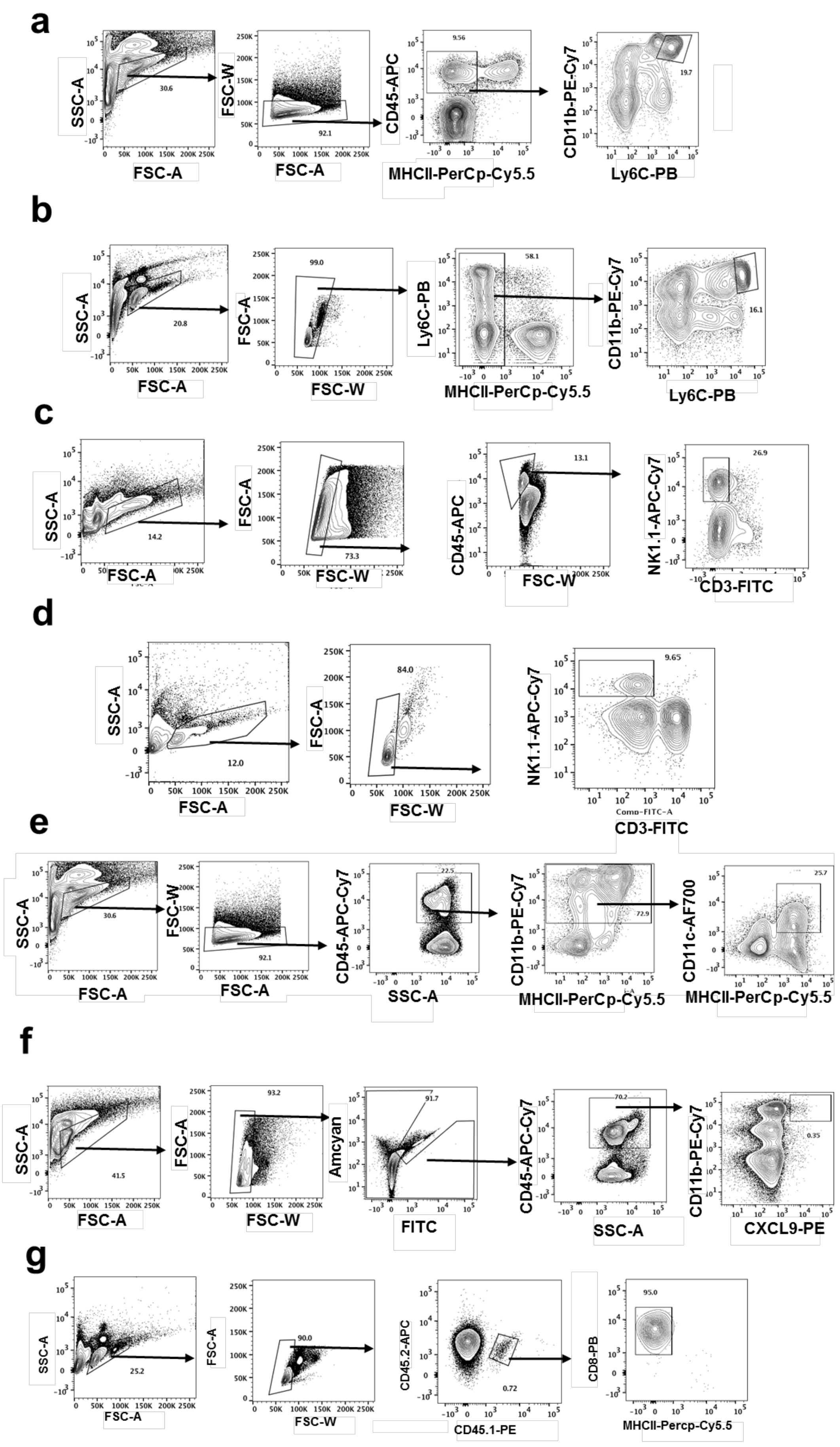

a
SSC-A
FSC-A
30.6
FSC-W
FSC-A
92.1
CD45-APC
MHCII-PerCp-Cy5.5
9.56
CD11b-PE-Cy7
Ly6C-PB
19.7
b
SSC-A
FSC-A
20.8
FSC-A
FSC-W
99.0
Ly6C-PB
MHCII-PerCp-Cy5.5
58.1
CD11b-PE-Cy7
Ly6C-PB
16.1
c
SSC-A
FSC-A
14.2
FSC-A
FSC-W
73.3
CD45-APC
FSC-W
13.1
NK1.1-APC-Cy7
CD3-FITC
26.9
d
SSC-A
FSC-A
12.0
FSC-A
FSC-W
84.0
NK1.1-APC-Cy7
Comp-FITC-A
CD3-FITC
9.65
e
SSC-A
FSC-A
30.6
FSC-W
FSC-A
92.1
CD45-APC-Cy7
SSC-A
22.5
CD11b-PE-Cy7
MHCII-PerCp-Cy5.5
72.9
CD11c-AF700
MHCII-PerCp-Cy5.5
25.7
f
SSC-A
FSC-A
41.5
FSC-A
FSC-W
93.2
Amcyan
FITC
91.7
CD45-APC-Cy7
SSC-A
70.2
CD11b-PE-Cy7
CXCL9-PE
0.35
g
SSC-A
FSC-A
25.2
FSC-A
FSC-W
90.0
CD45.2-APC
CD45.1-PE
0.72
CD8-PB
MHCII-Percp-Cy5.5
95.0

Supplementary Figure 16.

Other gating strategies. (**a**) An example of a gating strategy used to define the monocyte population in the FRT in Figures 5b, 8f, 8i and Supplementary Figures 9a, 10c and 12d. After gating on the live and single cells based on SSC-A, FCS-A and FSC-W, the CD45^{+} MHCII^{-} population was further examined and monocytes were gated as CD11b^{+}Ly6C^{hi}cells. (**b**) Gating strategy used to define the monocyte population in blood in Supplementary Figures 10a and 12c. Within the live, single cell and MHCII^{-} gates, monocytes were gated as CD11b^{+}Ly6C^{+} cells. (**c**) Gating strategy used to define NK cells in the FRT in Figure 6b and Supplementary Figures 10d and 12b. After gating on the live and single cells based on SSC-A, FCS-A and FSC-W, CD45^{+} cells were further examined for the expression of CD3 and NK1.1. (**d**) Gating strategy used to define NK cell population in blood in Figure 6a and Supplementary figures 10b and 12a. Within live single cell gates, NK cells were gated as NK1.1^{+}CD3^{-} cells. (**e**) Gating strategy used to define DCs in the FRT in Figure 5c. After gating on the live and single cells based on SSC-A, FCS-A and FSC-W, CD45^{+} cells were further divided into CD11b^{+} and CD11b^{-} populations. From the CD11b^{+} gate, DCs were gated as CD11c^{+}MHCII^{+}cells. (**f**) Gating strategy used to define CXC9-expressing cells in lungs in Supplementary Figure 8a. After gating on the live and single cells based on SSC-A, FCS-A and FSC-W, CD45^{+} cells are further examined for the expression of CXCL9 and CD11b. (**g**) Gating strategy corresponding to data in Figure 4c-e. Adoptively transferred CD45.1^{+} OT-I cells were identified based on CD45.1 and CD45.2 expression. They were further verified as CD8^{+}MHCII^{-}.

Supplementary Table 1. Primers used for either cloning or real time PCR

Gene	Forward Primer	Reverse Primer
HIV-1 CN54 gag	aaagcggccgcgttagttctctctaaaagg	tttctcgagctttaagcttgatatcg
Ad5	caacaagtcaacggatgtgg	ccgggctgtagtcattgttt

Supplementary Table 2. Antibodies used for Flow Cytometry.

Mouse Antigen	Clone	Conjugates	Source	Dilution
CD16/32 (FcRII/III)	2.4G2	unconjugated	BD Biosciences	1:50
CD8	53–6.7	PE-Cy7	BD Biosciences	1:250
CD4	RM4-5	PerCP	BD Biosciences	1:250
CD11b	M1/70	PE-Cy7	BD Biosciences	1:250
CD3	145-2C11	FITC	BD Biosciences	1:50
MHC class II	2G9	Biotin/Streptavidin	BD Biosciences	1:2000/1:1000
Gr-1	RB6-8C5	PerCP-Cy5.5	BD Biosciences	1:200
CD45R/B220	RA3-6B2	Biotin/ Streptavidin	BD Biosciences	1:250
CD4	GK1.5	Biotin/Streptavidin	BD Biosciences	1:250/1:1000
NKp46	29A1.4	APC	Biolegend	1:200
CD49b	DX5	Pacific Blue	Biolegend	1:200
CCR1	S15040E	APC	Biolegend	1:100
CCR5	HM-CCR5	PE-Cy7	Biolegend	1:100
CCR6	29-2L17	PE	Biolegend	1:100
CCR7	4B12	APC	eBioscience	1:100
CCR9	242503	FITC	R&D	1:100
CCR10	248918	PE	R&D	1:100
CXCR3	CXCR3-173	PE-Cy7	Biolegend	1:200
CXCR6	SA051D1	FITC	Biolegend	1:100
CXCL9	MIG-2F5.5	PE	Biolegend	1:100
α4β7	DATK32	PE	Biolegend	1:100
IFNg	XMG1.2	APC	eBioscience	1:200
Granzyme B	NGZB	PE-Cy7	eBioscience	1:100
CD11a	M17/4	FITC	eBioscience	1:200
MHC II	M5/114.15.2	PE	Biolegend	1:2000
MHC II	M5/114.15.2	PerCP	Biolegend	1:2000
Ly6C	HK1.4	eFluor® 450	eBioscience	1:200
NK1.1	PK136	APC-Cy7	Biolegend	1:200
CD11c	HL3	APC	BD Biosciences	1:250
CD69	H1.2F3	PE	eBioscience	1:200
CD45.1	A20	PE	Biolegend	1:200
CD45.2	104	APC	Biolegend	1:200
LIVE/DEAD Fixable Cell Stain Kit	Cat #: L34959	Yellow	Invitrogen	1:500
LIVE/DEAD Fixable Cell Stain Kit	Cat #: L10119	Near-IR	Invitrogen	1:50
Tetramer	Db/HIV-1-CN54 gag 308-318	PE, APC and Brilliant Violet 421	NIH tetramer facility, Emory University	1:400
