# Supplementary material for: Skin immunisation activates an innate lymphoid cell-monocyte axis regulating CD8+ effector recruitment to mucosal tissues
Source: Nat Commun. 2019 May 17;10:2214. doi: 10.1038/s41467-019-09969-2 (PMC6525176; doi:10.1038/s41467-019-09969-2)
Supplement: Supplementary file 3 — Reporting Summary [file 41467_2019_9969_MOESM3_ESM.pdf]

nature research

Corresponding author(s): Linda S Klavinskis

Reporting Summary

Nature Research wishes to improve the reproducibility of the work that we publish. This form provides structure for consistency and transparency in reporting. For further information on Nature Research policies, see Authors & Referees and the Editorial Policy Checklist.

Statistical parameters

When statistical analyses are reported, confirm that the following items are present in the relevant location (e.g. figure legend, table legend, main text, or Methods section).

n/a	Confirmed	
☐	☒	The exact sample size (n) for each experimental group/condition, given as a discrete number and unit of measurement
☐	☒	An indication of whether measurements were taken from distinct samples or whether the same sample was measured repeatedly
☐	☒	The statistical test(s) used AND whether they are one- or two-sided<br>*Only common tests should be described solely by name; describe more complex techniques in the Methods section.*
☒	☐	A description of all covariates tested
☐	☒	A description of any assumptions or corrections, such as tests of normality and adjustment for multiple comparisons
☐	☒	A full description of the statistics including central tendency (e.g. means) or other basic estimates (e.g. regression coefficient) AND variation (e.g. standard deviation) or associated estimates of uncertainty (e.g. confidence intervals)
☐	☒	For null hypothesis testing, the test statistic (e.g. F, t, r) with confidence intervals, effect sizes, degrees of freedom and P value noted<br>*Give P values as exact values whenever suitable.*
☒	☐	For Bayesian analysis, information on the choice of priors and Markov chain Monte Carlo settings
☒	☐	For hierarchical and complex designs, identification of the appropriate level for tests and full reporting of outcomes
☒	☐	Estimates of effect sizes (e.g. Cohen's d, Pearson's r), indicating how they were calculated
☐	☒	Clearly defined error bars<br>*State explicitly what error bars represent (e.g. SD, SE, CI)*

Our web collection on statistics for biologists may be useful.

Software and code

Policy information about availability of computer code

Data collection	Flow cytometry data were collected using BD FACSDiva™ software.
Data analysis	• Flow cytometry data analysis was performed using FlowJo software version 9.7.5 (Tree Star) for Mac<br>• Statistical analysis between groups was performed with Graph pad prism version 6

For manuscripts utilizing custom algorithms or software that are central to the research but not yet described in published literature, software must be made available to editors/reviewers upon request. We strongly encourage code deposition in a community repository (e.g. GitHub). See the Nature Research guidelines for submitting code & software for further information.

Data

Policy information about availability of data

All manuscripts must include a data availability statement. This statement should provide the following information, where applicable:

- Accession codes, unique identifiers, or web links for publicly available datasets
- A list of figures that have associated raw data
- A description of any restrictions on data availability

The data that support the findings of this study are available from the corresponding author on request.

Field-specific reporting

Please select the best fit for your research. If you are not sure, read the appropriate sections before making your selection.

☒ Life sciences ☐ Behavioural & social sciences ☐ Ecological, evolutionary & environmental sciences

For a reference copy of the document with all sections, see nature.com/authors/policies/ReportingSummary-flat.pdf

Life sciences study design

All studies must disclose on these points even when the disclosure is negative.

Sample size	No sample-size calculations were performed. Sample size was determined to be adequate based on the magnitude and consistency of measurable differences between groups.
Data exclusions	Samples were not excluded from the flow cytometry analyses unless a severe anomaly was detected in the cell size gate (which occured in two samples).
Replication	All data was successfully replicated at least two times.<br>How many times each experiment was performed and which statistical analysis was used is indicated in the figure legends.
Randomization	Mice were age and sex matched. Mice were allocated to groups by an independent animal caretaker.
Blinding	Investigators were not blinded to mouse genotypes during experiments. Data reported for mouse experiments are not subjective but rather based on quantitative flow cytometry.<br>For quantitative PCR analysis tissue samples were coded and sent to a different laboratory for assay and analysis, for virus challenge experiments samples were coded for assay and analysis.

Reporting for specific materials, systems and methods

Materials & experimental systems

n/a	Involved in the study
☐	☒ Unique biological materials
☐	☒ Antibodies
☐	☒ Eukaryotic cell lines
☒	☐ Palaeontology
☐	☒ Animals and other organisms
☒	☐ Human research participants

Methods

n/a	Involved in the study
☒	☐ ChIP-seq
☐	☒ Flow cytometry
☒	☐ MRI-based neuroimaging

Unique biological materials

Policy information about availability of materials

Obtaining unique materials	• Recombinant vaccinia virus encoding HIV-1 CN54 gag was generated by us in this study. No restriction is placed on availability, where a reasonable request is made.<br>• Tetramer Db HIV-1-CN54 gag 308-318 was synthesised by the NIH Tetramer core facility (Emory University) and would be available by request from the NIH Tetramer core (Emory University).

Antibodies

Antibodies used	Antibodies are listed below as: target label (clone, company, catalog number, dilution)<br><br>CD16/32 (FcRII/III) purified, unconjugated (clone 2.4G2, BD Biosciences, 553141) 1:50<br>CD8 PE-Cy7 (clone 53–6.7, BD Biosciences, 552877) 1:250<br>CD4 PerCP (clone RM4-5, BD Biosciences, 553052) 1:250<br>CD11b PE-Cy7 (clone M1/70, BD Biosciences, 561098) 1:250<br>CD3 FITC (clone 145-2C11, BD Biosciences, 553061) 1:50<br>MHC class II Biotin/Streptavidin (clone 2G9, BD Biosciences, 553622) 1:2000/1:1000<br>Gr-1 PerCP-Cy5.5 (clone RB6-8C5, BD Biosciences, 552093) 1:200

CD45R/B220 Biotin/ Streptavidin (clone RA3-6B2, BD Biosciences, 553085) 1:250
CD4 Biotin/Streptavidin (clone GK1.5, BD Biosciences, 553728) 1:250/1:1000
NKp46 APC (clone 29A1.4, Biolegend, 137607) 1:200
CD49b Pacific Blue (clone DX5, Biolegend, 108917) 1:200
CCR1 APC (clone S15040E, Biolegend, 152504) 1:100
CCR5 PE-Cy7 (clone HM-CCR5, Biolegend, 107017) 1:100
CCR6 PE (clone 29-2L17, Biolegend, 129821) 1:100
CCR7 APC (clone 4B12, eBioscience, 47-1971-80) 1:100
CCR9 FITC (clone 242503, R&D Biosystems, FAB2160F) 1:100
CCR10 PE (clone 248918, R&D Biosystems, FAB2815P) 1:100
CXCR3 PE-Cy7 (clone CXCR3-173, Biolegend, 126515) 1:200
CXCR6 FITC (clone SA051D1, Biolegend, 151107) 1:100
CXCL9 PE (clone MIG-2F5.5, Biolegend, 515603) 1:100
α4β7 PE (clone DATK32, Biolegend, 120605) 1:100
IFNg APC (clone XMG1.2, eBioscience, 17-7311-82) 1:200
Granzyme B PE-Cy7 (clone NGZB, eBioscience, 25-8898-82) 1:100
CD11a FITC (clone M17/4, eBioscience, 11-0111-82) 1:200
MHC II PE (M5/114.15.2, Biolegend, 107607) 1:2000
MHC II PerCP (M5/114.15.2, Biolegend, 107623) 1:2000
Ly6C eFluor® 450 (clone HK1.4, eBioscience, 48-5932-80) 1:200
NK1.1 APC-Cy7 (clone PK136, Biolegend, 108723) 1:200
CD11c APC (clone HL3, BD Biosciences, 561119) 1:250
CD69 PE (clone H1.2F3, ebioscience, 12-0691-81) 1:200
CD45.1 PE (clone A20, Biolegend, 110707) 1:200
CD45.2 APC (clone 104, Biolegend, 109813) 1:200
LIVE/DEAD Fixable Yellow Dead Cell Stain Kit (Invitrogen, L34959) 1:500
LIVE/DEAD Fixable Near-IR Dead Cell Stain Kit (Invitrogen, L10119) 1:50
Mouse IgG2a isotype control unconjugated (clone C1.18.4, InVivoMAb, BE0085-A050MG) 200ug
Rat IgG1 isotype control unconjugated (clone HRPN, InVivoMAb, BE0088) 200ug
CXCR3 unconjugated (clone CXCR3-173, InVivoMAb, BE0249) 200ug
IFNγ unconjugated (clone XMG1.2, InVivoMAb, BE0055) 500ug
NK1.1 unconjugated (clone PK136, InvivoMab, BE0036-A050MG) 200ug

Tetramer Db/HIV-1-CN54 gag 308-318, in PE, APC and Brilliant violet 421 were produced by the NIH tetramer facility, Emory University and were used at 1:400

Validation

The monoclonal antibodies listed above are standard reagents used in the field and validated in the literature as cited on the manufacturers websites, as well as by the manufacturers data sheets themselves. The tetramers were validated in-house and have been published previously.

Eukaryotic cell lines

Policy information about cell lines

Cell line source(s)

• BHK- Baby Hamster Kidney 21, obtained from ATCC ((CRL12071)

• BSC-40- African green monkey kidney epithelial cells; obtained from ATCC (CCL26)

• RK13- Rabbit Kidney 13, obtained from ATCC (VR112)

Authentication

Authentication was provided by ATCC

Mycoplasma contamination

The cell lines were negative for mycoplasma prior to initiating experiments.

Commonly misidentified lines
(See ICLAC register)

No commonly misidentified cell lines were used.

Animals and other organisms

Policy information about studies involving animals; ARRIVE guidelines recommended for reporting animal research

Laboratory animals

Mice used in the experiments were 7-8 weeks old female mice.
C57BL/6 (B6 strain) Envigo.
B6.SJL CD45.1 from The Francis Crick Institute (London)
Rag1-/-, Laboratory of Graham Lord
Rag2-/-γcnull, Laboratory of Graham Lord

Wild animals

Study did not involve wild animals.

Field-collected samples | Study did not involve samples collected from the field.

Flow Cytometry

Plots

Confirm that:

- [x] The axis labels state the marker and fluorochrome used (e.g. CD4-FITC).
- [x] The axis scales are clearly visible. Include numbers along axes only for bottom left plot of group (a 'group' is an analysis of identical markers).
- [ ] All plots are contour plots with outliers or pseudocolor plots.
- [x] A numerical value for number of cells or percentage (with statistics) is provided.

Methodology

Sample preparation	Samples were prepared as described in the methods section. Briefly, animals were culled according to procedures approved by the UK home office. All tissues were harvested following perfusion. Single cell suspensions were prepared from spleen and LN's by mashing tissues through a 70uM sieve. Single cell suspensions from liver, lung and female reproductive tract tissue were obtained by enzymatic digestion with Collagenase D (RocheTM) and DNase I from bovine pancreas (RocheTM) as described in the methods section, followed by disruption with a pipette. All suspensions were run through a sieve. Erythrocytes were lysed using a hypotonic solution. Before staining with specific antibodies, cells were pre-treated with Fc block (clone 2.4G2, cat # 553142, BD Biosciences). Live/Dead stains were used to exclude dead cells.
Instrument	BD FACSCanto II and BD LSR Fortessa™
Software	FlowJo software version 9.7.5 (Tree Star) for Mac
Cell population abundance	For all cell populations analysed, abundances are indicated in the figure plots.
Gating strategy	All gate strategies captured cells by FSC vs SSC area, single cells by FSC height versus area, and live cells by binding of cell a viability dye. Gating strategies beyond this differed by experiment. Figures exemplifying the gating strategy will be provided in the supplementary figures.

- [x] Tick this box to confirm that a figure exemplifying the gating strategy is provided in the Supplementary Information.
